# Adherence to emerging plant-based dietary patterns and its association with cardiovascular disease risk in a nationally representative sample of Canadian adults.

**DOI:** 10.23889/ijpds.v7i3.2104

**Published:** 2022-08-25

**Authors:** Svilena Lazarova, Jason Sutherland, Mahsa Jessri

**Affiliations:** 1 Food, Nutrition and Health Program, University of British Columbia, Vancouver, British Columbia, Canada; 2 Centre for Health Services and Policy Research, University of British Columbia

## Objectives

Little is known about the role of emerging plant-based dietary patterns in cardiovascular disease (CVD) at the national population level. The objectives of this research were to assess the validity and reliability of newly-established plant-based dietary indices, and to evaluate their associations with CVD risk among Canadian adults.

## Approach

Data were obtained from repeated 24-hour dietary recalls of adult participants in the cross-sectional, nationally representative Canadian Community Health Survey cycle 2004 linked to vital statistics (n=12,323) and cycle 2015 (n=14,026). Plant-based diet quality was assessed with a revised Plant-based Dietary Index (PDI), EAT-Lancet Reference Diet (ERD) score and the latest Dietary Guidelines for Americans Adherence Index (DGAI) 2020. Weighted multivariate analyses were used for testing associations between diet quality and lifestyle characteristics, and weighted multivariable-adjusted Cox proportional-hazards models for associations with CVD risk.

## Results

Construct validity was confirmed for the revised PDI and DGAI 2020 (but not ERD) as participants in the highest (healthiest) quartile, compared to those in the lowest (least healthy), were more likely to be female (52.63±1.27% compared to 44.8±1.65% for revised PDI; 59.37±2.01% compared to 40.84±1.71% for DGAI 2020), older (50.55±0.39 compared to 45.56±0.43 for revised PDI; 51.57±0.39 compared to 46.35±0.54 for DGAI 2020), to have post-secondary education (32.36±1.55% compared to 21.12±1.31% for revised PDI; 34.17±2.69% compared to 17.87±0.98% for DGAI 2020), and less likely to be daily smokers (8.21±1% compared to 17.06±1.45% for revised PDI; 7.36±1.71% compared to 21.53±1.58% for DGAI 2020) (P-trend<0.0001). No significant associations were observed between dietary index scores and CVD risk.

## Conclusion

Revised PDI and DGAI 2020 provided valid and meaningful measures of plant-based eating among Canadians, while validity of ERD was not directly confirmed. Adherence to the plant-based dietary patterns was not associated with CVD risk. Future large-scale analyses are necessary to further evaluate the role of plant-based eating in CVD prevention.

